# SAT1 promotes the progression of OA by regulating TRIM33-mediated p53 acetylation to enhance ferroptosis

**DOI:** 10.1371/journal.pone.0332761

**Published:** 2025-10-08

**Authors:** Jiexiang Yang, Jing Jiang, Jing Wang, Lin Luo

**Affiliations:** Department of Orthopedics, The Affiliated Traditional Chinese Medicine Hospital, Southwest Medical University, Luzhou, Sichuan Province, P.R. China; The Affiliated Changzhou No 2 People's Hospital of Nanjing Medical University, CHINA

## Abstract

**Background:**

Ferroptosis is a nonapoptotic form of cell death characterized by lipid peroxidation and intracellular iron accumulation. OA is a prevalent joint disease, and as OA progresses, inflammation or iron overload can induce ferroptosis in chondrocytes. However, research on genes that play important roles in this process remains insufficient.

**Methods:**

In this study, we identified the ferroptosis-related gene SAT1 by analyzing OA-associated GEO datasets. For *in vivo* experiments, we induced an OA mouse model by transecting the medial meniscus ligament. *In vitro*, we analyzed the biological functions of SAT1 in ATDC5 cells using Cell Counting Kit-8 (CCK-8), trypan blue staining, Western blot, and detection of ferroptosis-related indicators. Additionally, we explored the mechanisms underlying SAT1’s role in OA progression through immunoblotting, ubiquitination, and acetylation immunoprecipitation experiments.

**Results:**

Bioinformatics analysis revealed a close association between OA and ferroptosis. Our experimental results showed that overexpression of SAT1 effectively induced ferroptosis in ATDC5 cells. Mechanistically, SAT1 promoted p53 stability by downregulating TRIM33, which inhibits p53 acetylation. By suppressing TRIM33 expression, SAT1 enhanced p53 acetylation and stability, thereby increasing ferroptosis and exacerbating OA progression.

**Conclusion:**

In summary, our data indicate that SAT1 is a potential key gene in OA, revealing the crucial role of the SAT1-TRIM33-p53 axis in OA pathogenesis. This axis promotes ferroptosis by enhancing p53 acetylation, suggesting that targeting SAT1 may represent a novel therapeutic strategy for improving OA.

## Introduction

Osteoarthritis (OA), characterized by progressive cartilage degradation, is a leading cause of disability worldwide [1]. Accumulating evidence indicates that dysregulated cell death, including apoptosis, autophagy, and more recently, ferroptosis, plays a critical role in the loss of chondrocytes and the progression of OA [2]. Although the pathogenesis of OA is not fully understood, studies have shown that cellular senescence, gradual degeneration of the cartilage extracellular matrix (ECM), synovial inflammation, and abnormal apoptosis and survival patterns of chondrocytes play key roles in OA progression [3]. In recent years, multiple molecular mechanisms have been found to be closely related to the occurrence and development of OA, including oxidative stress, ferroptosis, abnormal activation of the Wnt signaling pathway, and miRNA regulatory imbalance [4–7]. Therefore, understanding the pathophysiological processes of OA at the molecular and cellular levels will help identify potential diagnostic biomarkers and therapeutic targets.SAT1 (Spermidine/Spermine N1-Acetyltransferase 1) is a key enzyme in polyamine metabolism, responsible for the acetylation of polyamines (such as spermidine and spermine), thereby regulating intracellular polyamine homeostasis and redox balance [8,9]. In addition to its classic role in polyamine metabolism, recent studies have found that SAT1 plays non-classical functions in various diseases, including epigenetic regulation, cell survival, and inflammatory responses. For example, in ovarian cancer, SAT1 promotes tumor cell anchorage-independent survival through histone H3K27 acetylation, driving peritoneal and pelvic metastasis [8]; in triple-negative breast cancer (TNBC), SAT1 stabilizes YBX1 protein and mTOR mRNA, inhibits autophagy, and promotes tumor progression [10]. In the field of OA, SAT1 has been shown to exacerbate chondrocyte damage by regulating ferroptosis and inflammatory responses, and its inhibition can alleviate OA progression [9]. Moreover, SAT1 is also closely related to chemotherapy resistance and metabolic reprogramming, making it a potential therapeutic target for multiple diseases [11,12]. These studies highlight the pleiotropic effects of SAT1 in diseases and provide important evidence for the development of targeted SAT1 therapeutic strategies.P53, a critical regulator of cell fate with well-established tumor-suppressive functions, has also been implicated in non-cancer pathologies, including OA. Acetylation by histone acetyltransferases (such as p300/CBP) at multiple lysine sites on p53 (such as K120, K373, K382) promotes its binding to DNA and activation of downstream target genes (such as p21, BAX), thereby regulating cell cycle arrest, apoptosis, and ferroptosis [13,14]. Recent studies have also found that p53 acetylation plays an important role in non-tumor diseases [15]. For example, in OA, chondrocyte senescence and inflammatory responses are closely related to elevated p53 acetylation levels, and engineered mesenchymal stem cell exosomes can restore the function of senescent chondrocytes by regulating the p53 signaling pathway [16]. In addition, in acute lung injury (ALI), KAT8-mediated p53 acetylation (K120) can activate the NLRP3 inflammasome, exacerbating alveolar damage, while inhibiting KAT8 or using its inhibitor MG149 can significantly reduce inflammation and oxidative stress [17]. These studies not only reveal the pleiotropic regulatory mechanisms of p53 acetylation in diseases but also provide new ideas for the development of targeted p53 acetylation therapeutic strategies.Ferroptosis is an iron-dependent form of regulated cell death characterized by intracellular iron accumulation, glutathione (GSH) depletion, mitochondrial dysfunction, and lipid peroxidation [18]. In recent years, the role of ferroptosis in various diseases has garnered increasing attention, including OA, cancer, cardiovascular diseases, and neurodegenerative disorders [19,20]. In OA, ferroptosis is closely associated with the pathologies of articular cartilage, subchondral bone, and synovial tissues, all of which are related to iron overload and oxidative stress. Studies have shown that inhibiting ferroptosis in chondrocytes can promote cell proliferation, delay extracellular matrix (ECM) degradation, and alleviate synovial hyperplasia and inflammatory responses [21]. Additionally, metformin can inhibit ferroptosis in chondrocytes by regulating the p53/SLC7A11 pathway, offering a new strategy for OA treatment [22]. Beyond OA, breakthroughs in cancer research related to ferroptosis, such as the mechanism of ferroptosis propagation mediated by Galectin-13, provide new targets for cancer therapy [23]. In liver diseases, ferroptosis is closely related to non-alcoholic fatty liver disease (NAFLD), liver fibrosis, and hepatocellular carcinoma (HCC), and targeting GPX4 or ACSL4 may become a potential intervention [24]. These research advancements indicate that ferroptosis plays a significant role in the occurrence and development of various diseases, and in-depth study of its mechanisms and regulatory pathways is expected to provide new strategies and targets for the treatment of related diseases.In this study, we revealed the key role of the SAT1-TRIM33-p53 regulatory axis in ferroptosis during OA. SAT1 promotes p53 protein stability by downregulating TRIM33 expression, thereby enhancing p53 acetylation levels, ultimately leading to chondrocyte ferroptosis and OA progression. This finding not only clarifies the new function of the polyamine metabolic enzyme SAT1 in OA but also establishes for the first time the molecular connection between TRIM33-p53 acetylation and ferroptosis. More importantly, this mechanism provides a theoretical basis for the development of OA treatment strategies targeting the SAT1/p53 pathway.

## Methods

Cell Culture and ReagentsATDC5 cells were obtained from Wuhan Pricella Biotechnology Co., Ltd. and maintained in Dulbecco’s Modified Eagle Medium (DMEM) (Gibco, Thermo Fisher Scientific, Cat. No. 11965–092) supplemented with 10% fetal bovine serum (FBS) (Gibco, Thermo Fisher Scientific, Lot No. A5669701), 1% penicillin-streptomycin solution (Gibco, Thermo Fisher Scientific, Cat. No. 15140–122), and 1% L-glutamine (Gibco, Thermo Fisher Scientific, Cat. No. 25030–081). Cells were incubated at 37 °C in a humidified atmosphere containing 5% CO_2_. The cell line was authenticated and tested for mycoplasma contamination before use.Mouse OA ModelThis study was approved by the Animal Ethics Committee of The Affiliated Traditional Chinese Medicine Hospital of Southwest Medical University and adhered to ARRIVE guidelines and NIH standards for laboratory animal care. Twenty-eight 8-week-old male C57BL/6J mice were randomly assigned to OA and sham surgery groups (n = 14 each) and housed under SPF conditions (22 ± 1°C, 55 ± 5% humidity) for 12 weeks. OA was induced by medial meniscal ligament transection, while sham operations followed identical procedures without ligament transection [25]. All surgical procedures were performed under sterile conditions by trained personnel, and anesthesia and analgesia protocols were strictly followed in accordance with institutional guidelines. Osteoarthritis was surgically induced by destabilization of the medial meniscus (DMM) via transection of the medial meniscotibial ligament. Briefly, a 3–4 mm longitudinal skin incision was made on the medial aspect of the knee under sterile conditions. The joint capsule was exposed by blunt dissection between the quadriceps and patellar tendon, followed by medial patellar dislocation. The medial meniscotibial ligament was then carefully transected using microscissors (Dumont #5). After ligament resection, the patella was repositioned, and the joint capsule was closed with a single 8–0 Prolene suture. The skin incision was closed with interrupted 6–0 nylon sutures (typically 2–3 stitches). Sham-operated mice underwent identical surgical exposure without ligament transection. Postoperative analgesia (meloxicam, 5 mg/kg/day) was administered for 72 hours. Prespecified humane endpoints included: ① > 20% body weight loss within 48 hours; ② Inability to access food/water autonomously; ③ Lameness score ≥3; ④ Persistent lethargy (>2 hours); or ⑤ Ulcerative infection at the surgical site. Any animal meeting criteria was euthanized within 1 hour (intravenous pentobarbital sodium). Surgical anesthesia was maintained with isoflurane (4% induction, 1.5% maintenance), with postoperative analgesia (meloxicam, 5 mg/kg/day) administered routinely for 72 hours and buprenorphine (0.05 mg/kg) supplemented as needed. Daily monitoring included body weight and mobility assessments, wound evaluations every other day, and weekly gait analysis. All personnel completed certifications in rodent aseptic surgery, pain recognition, and euthanasia techniques. All surviving animals were euthanized as planned at endpoint (OA:14, Sham:14) with no unscheduled mortalities.*DNA Constructs, shRNA, and Transfection Protocol*DNA ConstructsFor overexpression experiments, the full-length coding sequence of the SAT1 gene was amplified from a cDNA library and inserted into an expression vector (pcDNA3.1) with a Flag tag using restriction enzymes and ligase. The constructed vectors were verified for sequence accuracy by sequencing.shRNAshRNA was designed according to the literature [9,26]. Lipofectamine 3000 transfection reagent (Invitrogen^TM^, L3000015) was used to transfect shRNA into cervical cancer cells to achieve efficient gene knockdown. Short hairpin RNAs (shRNAs) targeting mouse SAT1 were designed and cloned into the pLKO.1 vector. The specific target sequence for SAT1 shRNA was 5’-GCTTTGGCATAGGATCAGAAA-3’. A non-targeting scramble sequence (5′-UUCUCCGAACGUGUCACGUTT-3′) was used as a negative control.TransfectionTransfection was performed when the cell confluency reached 60%−70%. According to the transfection reagent manual, shRNA or DNA constructs were mixed with the transfection reagent and added to the cells. After 6–8 hours of transfection, the medium was replaced with fresh culture medium and the cells were continued to be cultured. Cells were collected at 24, 48, and 72 hours post-transfection for subsequent assays.

RNA Isolation, RT-PCR, and RT-qPCRTotal RNA was isolated from cultured cells using TRIzol reagent (Invitrogen^TM^, 15596026CN), followed by cDNA synthesis using the PrimeScript RT Master Mix (TaKaRa, RR036Q). qPCR was performed using the Applied Biosystems QuantStudio 5 system with Takara SYBR Green Master Mix. The reaction mixture (20 μL) contained 10 μL 2 × Master Mix, 0.4 μL ROX Reference Dye II (50×), 0.8 μL of each primer (10 μM), 2 μL cDNA, and 6 μL nuclease-free water. The cycling conditions were: 95°C for 30 sec, followed by 40 cycles of 95°C for 5 sec and 60°C for 30 sec. Melt curve analysis was performed to verify amplification specificity. Primer sequences are provided in Supplemental Table 1. All reactions were run in triplicate. No-template controls and reverse transcription controls were included. Amplification efficiencies were validated by standard curves, and GAPDH was confirmed as a stable reference gene. Western Blot AnalysisAfter treating ATDC5 cells with various methods, the cells were collected, washed with cold PBS (Sigma Aldrich, P5493), and incubated with RIPA lysis buffer (Millipore, 20−188) at 4 °C for 30 minutes to extract total protein. Protein concentration was determined using the BCA protein assay (Sigma Aldrich, BCA1), and equal amounts of protein were loaded and separated by electrophoresis on SDS-PAGE gels, then transferred to PVDF membranes. The membranes were blocked with 5% BSA powder and incubated overnight with primary antibodies at 4 °C, followed by incubation with HRP-conjugated secondary antibodies for 1−2 hours. Finally, proteins were detected by chemiluminescence (ECL). Immunoblotting was performed on protein samples using Anti-SAT1 antibody (Abcam, ab105220, 1:1000), Anti-p53 antibody [PAb 240] (Abcam, ab26, 1:10000), recombinant Anti-TRIM33 antibody [EPR25102−19] (Abcam, ab300146, 1:1000), Anti-beta Actin antibody [mAbcam 8226] (Abcam, ab8226, 1:5000), and Acetyl-p53 (Lys379) Antibody (Cell Signaling Technology, #2570, 1:1000).In Vivo Ubiquitination and CHX Chase AssayCells were transiently transfected with the specified plasmids for 48 hours, followed by treatment with the proteasome inhibitor MG132 (MedChemExpress, HY-13259) at a concentration of 10 μM for 6 hours. Subsequently, cells were lysed in denaturing buffer and subjected to immunoprecipitation (IP) analysis. The half-life of proteins was assessed using the cycloheximide (CHX) chase assay. Briefly, cells were treated with CHX (100 μg/ml) (MedChemExpress, HY-12320), and immunoblotting was performed at designated time points post-treatment. To identify the pathway of protein degradation, cells were co-incubated with the proteasome inhibitor MG132 at 10 μM, followed by immunoblotting analysis at specified time intervals.CCK-8 AssayThree thousand cells were seeded in 200 μl of culture medium into a 96-well plate, and measurements were taken 1, 3, 5, and 7 days post-seeding according to the manufacturer’s protocol. The cells were incubated in a 100 μl reaction mixture (10 μL CCK-8 (Sigma Aldrich, 96992) and 90 μL DMEM) for 2 hours, and absorbance was measured at a wavelength of 450 nm. For each experimental condition, measurements were performed in six technical replicates per experiment. Data were collected from three independent biological experiments conducted on different days using freshly prepared cells. Results are presented as the mean ± standard deviation (SD) of these independent replicates.EdU AssayStable transfected cell lines were seeded into 96-well plates (2 × 10^4^ cells per well) and cultured in DMEM for one day. Subsequently, the cells were incubated with 50 μmol/L EdU (RiboBio, C10310-2) at 37 °C for 2 hours. The cells were then fixed with a 4% formaldehyde solution for 30 minutes, followed by permeabilization with 0.5% Triton X-100 for 10 minutes. After adding 400 μL of 1 × Apollo reaction mixture, 400 μL of Hoechst 33342 was also added. The cells were rinsed three times with PBS before observing EdU-positive cells. Finally, images of the cells were captured using a microscope.*Lipid ROS Generation Analysis*Cells were rinsed once with PBS, and then incubated with PBS containing 10 μM C11-BODIPY (581/591) (#D3861, Thermo Fisher Scientific) at 37 °C in a cell culture incubator for 30 minutes. The working solution was discarded, and the cells were washed twice with PBS, followed by observation of the fluorescence signal under a fluorescence microscope.*Iron Content Assay*The relative iron concentration in cell lysates was assessed using the Iron Assay Kit (Colorimetric) (ab83366) according to the manufacturer’s instructions. In brief, cells were seeded in six-well plates and homogenized with five volumes of buffer. After centrifugation at 13,000 g for 15 minutes at 4 °C to remove insoluble material, the iron reducing agent was added to the sample wells. The buffer was gently mixed and then allowed to react in the dark at 37 °C for 30 minutes. Subsequently, 100 μL of the iron probe was added to each sample, and the mixture was allowed to react in the dark at room temperature for 1 hour. Finally, the absorbance was measured at 593 nm using a microplate reader.*Glutathione (GSH) and Malondialdehyde (MDA) Assay*This experiment employed the Glutathione Peroxidase Assay Kit (Colorimetric) (ab102530) and the Lipid Peroxidation (MDA) Assay Kit (S0131S). Cells in six-well plates were treated with or without 5 μM of Erastin for 24 hours, then washed once with PBS, and centrifuged at 800 rpm for 5 minutes to collect the supernatant. Next, 30 μl of the protein removal reagent solution was mixed with the cell pellet at a volume three times that of the cell precipitate. After thorough vortexing, the samples were subjected to two freeze-thaw cycles in liquid nitrogen and a 37 °C water bath. After placing on ice for 5 minutes and centrifuging at 4 °C at 10,000 g for 10 minutes, the supernatant was used for the determination of total glutathione. Samples should be temporarily stored at 4 °C, and those not determined immediately should be stored at −70 °C for up to 10 days. Finally, the absorbance was measured at 405 nm using a microplate reader.Enzyme-linked immunosorbent assay (ELISA) Mouse serum was obtained by centrifugation at 300 rpm for 10 minutes. Mouse serum was centrifuged at 300 rpm for 10 minutes and tested using the YOBIBIO (Shanghai, China) Mouse Serum IL-1β and TNF-α Assay Kit according to the manufacturer’s instructions.*Micro-CT Scanning*After the removal of skin and muscle, the entire right knee joint of the mouse was fixed with 4% paraformaldehyde. Subsequently, Micro-CT scanning was performed to obtain the raw images. Selected areas of the raw images were reconstructed using three-dimensional reconstruction software NRecon. The region of interest (ROI) was analyzed using CTAnalyser. After setting uniform parameters, the software calculated the total volume (TV), bone volume (BV), volume ratio (BV/TV), degree of osteophyte maturation, size of osteophyte, and the thickness of the subchondral bone plate.Histology and ImmunohistochemistryAfter obtaining OA tissue samples, they were immediately fixed in 10% formalin at 4 °C overnight. The samples were then sent to the pathology department of XX Hospital for paraffin embedding, hematoxylin and eosin (HE) staining, and immunohistochemical (IHC) staining. The antibodies used for IHC were Anti-4 Hydroxynonenal antibody (ab46545, 1:50) and anti-cleaved Caspase-3 (ZRB1221, 1:50).*Bioinformatics Analysis*The GEO datasets were obtained from publicly available gene expression datasets (GSE55457, GSE114007, GSE55235, and GSE206848). The KOBAS database (http://kobas.cbi.pku.edu.cn/) was utilized to identify the interacting proteins of the target protein and the KEGG pathways of differentially expressed genes. Statistical AnalysisTo ensure the reliability of the research results, the data were derived from at least three independent experiments. Statistical analysis was performed using GraphPad Prism software (version 9.0.0). For comparisons between two groups, paired or unpaired two-tailed Student’s t-tests were used. When comparing more than two groups, one-way analysis of variance (ANOVA) followed by multiple comparisons was employed. In experiments involving two factors, comparisons among four or more groups were conducted using two-way ANOVA followed by multiple comparisons. A *p < 0.05 level was considered statistically significant.

## Results

### SAT1 involved in polyamine metabolism is associated with ferroptosis

**Fig 1:**
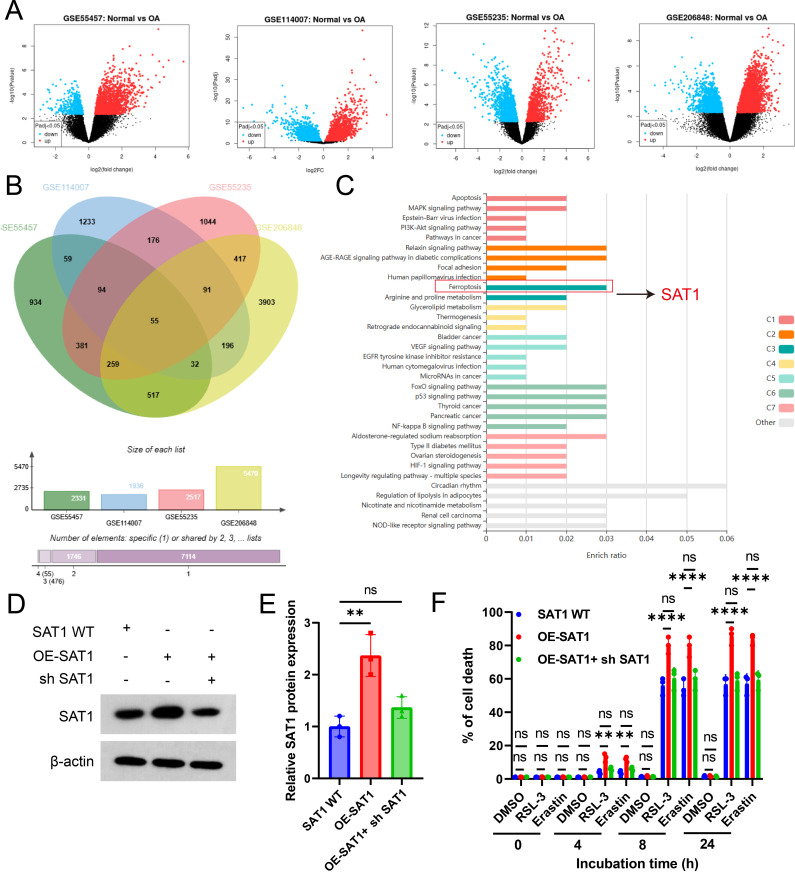
Bioinformatics analysis indicated that SAT1 is involved in ferroptosis. **(A)** Analysis of differentially expressed genes in GEO datasets related to OA. **(B)** Venn diagram of common DEGs among the GSE55457, GSE114007, GSE55235 and GSE206848 datasets. **(C)** Top significant pathways for overlapped differentially expressed genes in OA by Kyoto Encyclopedia of Genes and Genomes (KEGG) pathway enrichment analysis. **(D-E)** Western blot analysis of SAT1 protein expression levels in ATDC5 cells treated with SAT1 wild-type (WT), SAT1 overexpression (OE-SAT1), and SAT1 knockout (KO) constructs. The corresponding quantitative result is presented in **(E)**. **(F)** EDU assay of SAT1 WT, KO and OE ATDC5 cells viability over time ± Erastin 5 μM or RSL3 5 μM. **(G)** Trypan blue staining cell death in SAT1 WT, KO and OE ATDC5 cells over time ± Erastin 5 μM or RSL3 5 μM. Error bars represent ± / + SEM unless indicated otherwise. *p < 0.05, **p < 0.01, ***p < 0.001,****p < 0.0001.

To identify potential therapeutic targets for OA, we retrieved four datasets from the Gene Expression Omnibus (GEO) database. By employing a Venn intersection analysis, we identified significantly differentially expressed genes (DEGs) in OA (Fig 1A-B). Subsequent KEGG pathway analysis of these DEGs revealed an association between OA and ferroptosis, leading to the identification of SAT1, an enzyme responsible for the acetylation of polyamine compounds (Fig 1C). To validate the bioinformatics prediction that SAT1 is involved in the regulation of ferroptosis, we generated ATDC5 cell lines with stable knockdown or overexpression of SAT1 (Fig 1D-E). Cells were then treated with ferroptosis activators (RSL3) or inducers (Erastin) [27,28]. Our experimental results demonstrated that cell mortality increased with incubation time. Moreover, overexpression of SAT1 enhanced cell mortality, whereas SAT1 knockdown reduced it (Fig 1F-G). Collectively, these findings suggest that SAT1 may play a role in the regulation of ferroptosis.

### Overexpression of SAT1 Promotes Ferroptosis *In Vitro*

**Fig 2:**
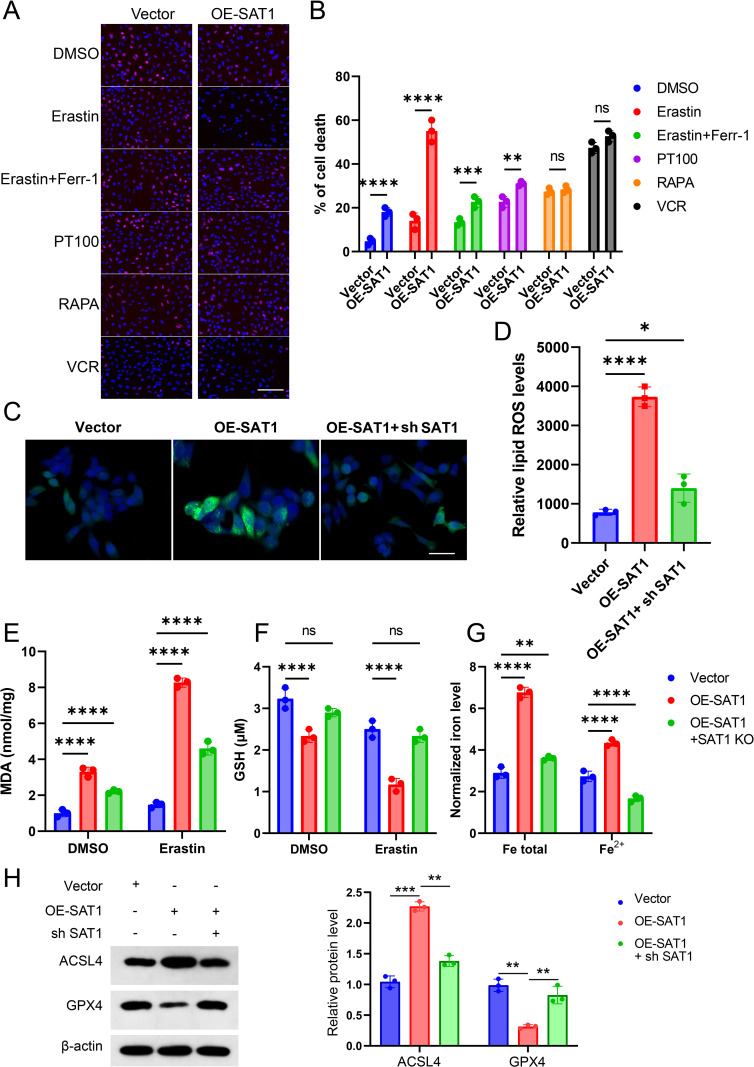
Overexpression of SAT1 promotes ferroptosis in vitro. **(A)** EDU assay of Vector and OE-SAT1 ATDC5 cells incubated with DMSO or Erastin 5μM or Erastin 5μM and Ferr-1 5μM or PT100 10μM or RAPA 10μM or VCR 2μM for 24 h**. (B)** Trypan blue staining cell death in Vector and OE-SAT1 ATDC5 cells treated with DMSO or Erastin 5 μM or Erastin 5 μM and ferr-1 5 μM or PT100 10 μM or RAPA 10 μM or VCR 2 μM for 24 h. **(C)** Lipid ROS analyzed by fluorescence microscope in Vector, OE-SAT1, and OE-SAT1 + AT1 KO ATDC5 cells. **(D)** Lipid ROS analyzed in Vector, OE-SAT1, and OE-SAT1 + SH SAT1. **(E)** MAD level was detected in Vector, OE-SAT1, and OE-SAT1 + SH SAT1, by treatment with Erastin 5 μM or not for 24 h. **(F)** GSH level was detected in Vector, OE-SAT1, and OE-SAT1 + SH SAT1, by treatment with Erastin 5 μM or not for 24 h. **(G)** Total intracellular iron and Fe2 + were detected in Vector, OE-SAT1, and OE-SAT1 + SH SAT1 ATDC5 cells. **(H)** Western blot analysis and quantification of ACSL4 and GPX4 protein levels in SAT1-overexpressing or SAT1-knockdown ATDC5 cells. β-actin served as a loading control. Error bars represent ± / + SEM unless indicated otherwise. *p < 0.05, **p < 0.01, ***p < 0.001,****p < 0.0001.

Consistent with the bioinformatics prediction mentioned above, we found that Erastin could induce more cell death in SAT1-overexpressing ATDC5 cells than PT-100 (a potent pyroptosis inducer), rapamycin (RAPA, a potent autophagy inducer), and vincristine (VCR, a potent apoptosis inducer). Moreover, the ferroptosis inhibitor ferrostatin-1 (Ferr-1) could reverse the cell death induced by Erastin. The EdU assay and cell viability staining with Trypan Blue also confirmed that the cell death induced by Erastin in SAT1-overexpressing ATDC5 cells could be rescued by the ferroptosis inhibitor (Fig 2A-B). In addition, we measured the levels of reactive oxygen species (ROS) at the cellular level. The experimental results showed that overexpression of SAT1 increased the levels of lipid ROS and malondialdehyde (MDA) in cells (Fig 2C-E) and decreased the level of glutathione (GSH) (Fig 2F). Subsequently, we depleted SAT1 in SAT1-overexpressing cells, and the levels of lipid ROS and MDA decreased (Fig 2C-E), while the level of GSH increased, especially when treated with Erastin (Fig 2F). Meanwhile, we detected ferrous ions (Fe^2+^) and total intracellular iron. Overexpression of SAT1 increased total intracellular iron and ferrous ions (Fe^2+^), while knockdown of SAT1 decreased them(Fig 2G). Consistently, Western blot analysis revealed that SAT1 overexpression increased ACSL4 expression and decreased GPX4 levels, while SAT1 knockdown reversed these effects (Fig 2H). These findings further confirm the role of SAT1 in promoting ferroptosis.In summary, these data indicate that overexpression of SAT1 leads to cellular lipid peroxidation and ferroptosis.

### Overexpression of SAT1 partially promotes the progression of OA via ferroptosis *in vivo*

**Fig 3:**
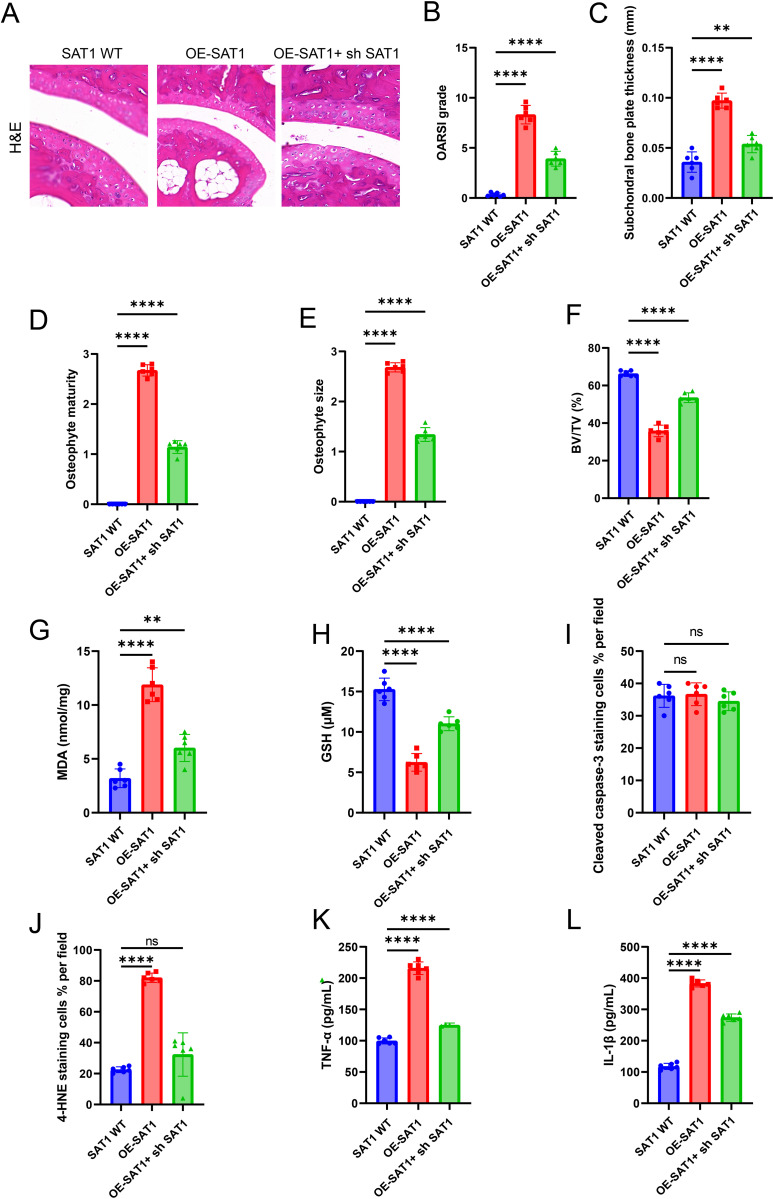
Overexpression of SAT1 promotes OA progression via ferroptosis *in vivo.* **(A)** Representative H&E staining of knee joint sections from sham, OA model, and treatment groups. Severe cartilage degradation and proteoglycan loss are observed in the OA model group, which is partially rescued by treatment. Scale bar: 200 μm. **(B)** OARSI scores for cartilage damage. **(C–F)** Micro-CT analysis showing bone volume fraction (BV/TV), osteophyte size and maturity, and subchondral bone plate thickness. **(G–H)** Measurement of MDA and GSH levels in joint tissues. **(I)** Immunohistochemical staining of cleaved caspase-3 in knee sections from SAT1 WT, OE-SAT1, and OE-SAT1 + shSAT1 groups. **(J)** Immunohistochemical staining of 4-HNE (a lipid peroxidation marker) in knee sections from SAT1 WT, OE-SAT1, and OE-SAT1 + shSAT1 groups. **(K–L)** ELISA analysis of serum IL-1β and TNF-α levels. Error bars represent ± / + SEM unless indicated otherwise. *p < 0.05, **p < 0.01, ***p < 0.001,****p < 0.0001.

To further investigate the effects of SAT1 and ferroptosis on the progression of OA, we established three experimental groups: SAT1 WT, OE-SAT1, and OE-SAT1 + SH SAT1. This design enabled us to observe the dynamic changes in cartilage degradation and evaluate the expression of related genes after surgery. Histological analysis using H&E staining revealed severe cartilage degradation and proteoglycan loss in the OA model group (OARSI score: 12.4 ± 1.8). In contrast, mice treated showed significant protection against cartilage damage (OARSI score: 6.2 ± 1.3, *p* = 0.003 vs. model grou*p*). Quantitative assessment of cartilage area confirmed a 52% reduction in the model group compared to sham controls (18.3 ± 2.1% vs. 38.1 ± 3.4%, *p* < 0.001), which was *p*artially reversed by treatment (30.5 ± 2.8%, *p* = 0.008 vs. model) (Fig 3A). The *in vitro* research results *p*rompted us to further study the impact of SAT1 on mouse OA. The OARSI score, which is commonly used to directly reflect the severity of cartilage damage, significantly increased in mice with SAT1 overexpression, but this condition could be reversed by knocking down SAT1 (Fig 3B). Micro-CT images showed that SAT1 overexpression significantly increased bone volume fraction, osteophyte size and maturity, and subchondral bone plate density. After SAT1 depletion, knee OA was significantly alleviated (Fig 3C-F). Meanwhile, SAT1 overexpression increased the MDA levels in tissues and decreased GSH levels, and these changes could be reversed by knocking down SAT1 (Fig 3G-H). Since ferroptosis is characterized by excessive lipid peroxidation, we performed immunohistochemical (IHC) analysis of 4-hydroxynonenal (4HNE), an indicator of ferroptosis, to characterize the lipid peroxidation levels in tissue samples of SAT1 wild-type (WT), overexpressing (OE), and knockout (KO) expressions. These results showed that compared with SAT1 WT cells, 4HNE staining increased in OE-SAT1 cells, and there were no changes in these lesions in apoptotic markers (Fig 3I-J). The ELISA detection system was used to detect systemic inflammatory responses. As expected, the levels of IL-1β and tumor necrosis factor-α (TNF-α) in the serum of mice with SAT1 overexpression significantly increased. Notably, knocking down SAT1 significantly reduced the release of these inflammatory cytokines IL-1β and TNF-α (Fig 3K-L). In summary, our data indicate that SAT1 partially promotes the development of OA by promoting ferroptosis.

### SAT1 functions as a p53 promoter to facilitate ferroptosis

**Fig 4:**
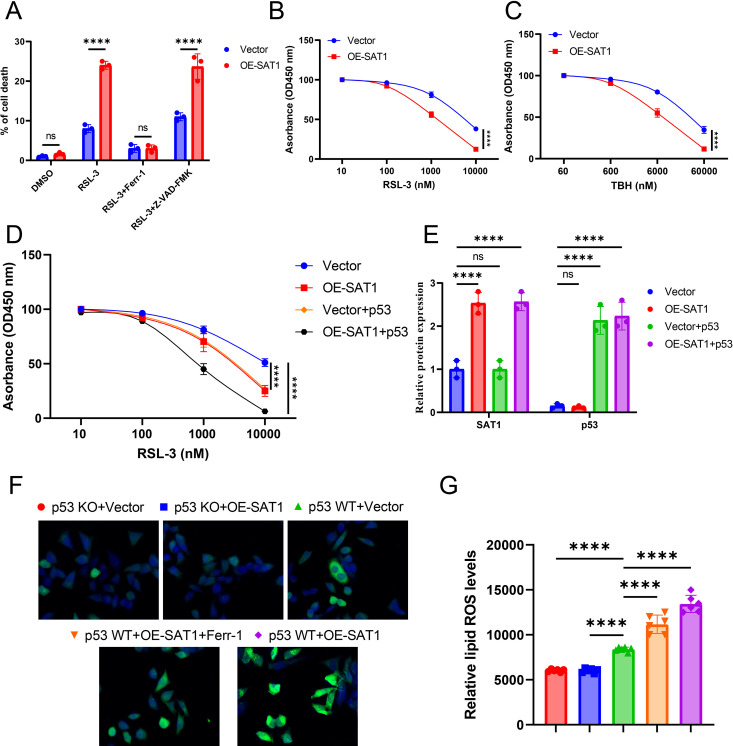
SAT1 promotes ferroptosis in a p53-dependent manner. (A) Trypan blue staining revealed cell death of Vector and OE-SAT1 ATDC5 cells treated with DMSO or RSL3 5 μM or RSL3 5 μM and Ferr-1 5 μM or RSL3 5 μM and Z-VAD-FMK 20 μM for 24 h. (*n* = 3 independent experiments). **(B)** CCK8 assay analyzed Vector and OE-SAT1 ATDC5 cells treated with DMSO or RSL3 in different concentration. (*n* = 3 independent experiments).(C) CCK8 assay analyzed Vector and OE-SAT1 ATDC5 cells treated with DMSO or TBH in different concentration. (*n* = 3 independent experiments). **(D)** CCK8 assay analyzed Vector and OE-SAT1with p53 transiently overexpressed ATDC5 cells treated with DMSO or RSL3 in different concentration. (*n* = 3 independent experiments).(E) The protein levels of indicated detected by Western blot in ATDC5 cells. (*n* = 3. independent experiments).(F-G) The lipid peroxidation level of subcutaneous tumor in indicated mice. Error bars represent ± / + SEM unless indicated otherwise. *p < 0.05, **p < 0.01, ***p < 0.001,****p < 0.0001.

To further explore the mechanism by which SAT1 promotes ferroptosis, we constructed ATDC5 cells stably overexpressing SAT1. We found that overexpression of SAT1 increased the cell death rate under RSL3 induction. This RSL3-induced cell death could be rescued by Ferr-1 (a ferroptosis inhibitor), but not by ZVAD-FMK (an apoptosis inhibitor), indicating that the mechanism specifically involves ferroptosis rather than apoptosis (Fig 4A). Further CCK-8 experiments revealed that ATDC5 cells overexpressing SAT1 exhibited higher sensitivity to cell death induced by RSL3 or tert-butyl hydroperoxide (TBH, a ROS inducer) (Fig 4B-C).In the study of cell death mechanisms, ferroptosis, as an iron-dependent form of cell death, is closely related to multiple intracellular regulatory factors. In recent years, the multifaceted role of p53 in the regulation of cell death has gradually been unveiled, and it may participate in the regulation of ferroptosis by affecting intracellular iron metabolism and the antioxidant system. Therefore, we introduced p53 to further investigate the relationship between p53 and ferroptosis. The experimental results showed that when p53 was introduced into ATDC5 cells, the sensitivity of cells overexpressing SAT1 to RSL3-induced cell death increased (Fig 4D-E). Moreover, at the tissue level, we observed the highest level of lipid peroxidation when SAT1 was overexpressed in the presence of p53. However, this effect was almost undetectable in p53 knockout cells. Meanwhile, Ferr-1 had a significant rescue effect on the lipid peroxidation levels in tissues overexpressing SAT1 and p53 (Fig 4F-G). Based on the above experimental results, we can conclude that SAT1 promotes ferroptosis in a p53-dependent manner.

### SAT1 Promotes Ferroptosis by Enhancing p53 Acetylation

**Fig 5:**
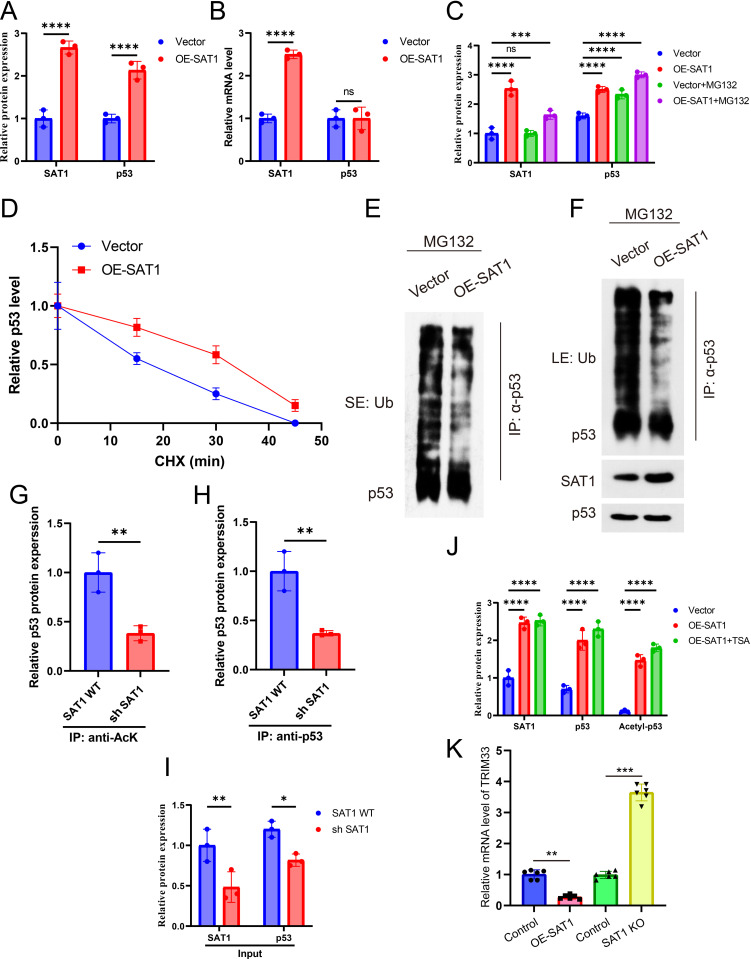
SAT1 promotes ferroptosis by enhancing p53 acetylation. (A) Western blot analysis of SAT1 and p53 protein levels in ATDC5 cells with or without SAT1 overexpression. (*n* = 3 independent experiments).(B) qPCR analysis of SAT1 and p53 mRNA levels in ATDC5 cells with or without SAT1 overexpression. (*n* = 3 independent experiments). **(C)** Vector and OE-SAT1 ATDC5 cells treated with MG132 or not (20 μM) for 6 h. (*n* = 3 independent experiments). **(D)** CHX 100 μg/mL pulsechase analysis of p53 protein levels in Vector and OE-SAT1 ATDC5 cells. (*n* = 3 independent experiments).(E-F) Vector and OE-SAT1 ATDC5 cells treated with MG132 (20 μM) for 6 h. Cell lysates collected for IP analysis. SEshort exposure, LE: long exposure. (*n* = 3 independent experiments). (G-I) Lysates from SAT1 WT and SH SAT1 ATDC5 cells loaded at a ratio of 1:2 was subjected to IP analysis. (*n* = 3 independent experiments). **(J)** Western blot analysis of SAT1, p53 and acetyl-p53 protein levels in ATDC5 Vector and OE-SAT1 cells treated with TSA 1 μM or not for 24 h. (*n* = 3 independent experiments). (K) qRT-PCR analysis of TRIM33 mRNA levels in ATDC5 cells with SAT1 overexpression or knockdown. GAPDH was used as an internal control.Error bars represent ± / + SEM unless indicated otherwise. *p < 0.05, **p < 0.01, ***p < 0.001,****p < 0.0001.

To investigate the regulatory effects of SAT1 on p53, we initially examined the impact of SAT1 on both p53 mRNA and protein levels. The findings revealed that overexpression of SAT1 significantly elevated p53 protein levels, whereas knocking down SAT1 reduced p53 protein levels (Fig 5A). However, p53 mRNA levels remained unchanged in response to either SAT1 knockdown or overexpression (Fig 5B), indicating that SAT1’s influence on p53 is mediated through post-translational modifications. Additionally, we observed that SAT1 extends the half-life of p53 and inhibits its ubiquitination process (Figs 5C-F). Given that acetylation is an indispensable modification mechanism for activating p53 in stress responses and ferroptosis [29,30], we further assessed the acetylation levels of p53. Using immunoprecipitation (IP) analysis with antibodies targeting pan-acetylated lysine (Pan-AcK) or p53, we examined lysates from control and SAT1 knockdown cells (containing equal amounts of p53). Compared to control cells, SAT1 knockdown cells exhibited a lower percentage of acetylated p53 (Figs 5G-I). In contrast, overexpression of SAT1 significantly increased p53 acetylation levels, comparable to the effect of HDAC inhibitor TSA in blocking p53 deacetylation (Fig 5J). Notably, qRT-PCR analysis showed that SAT1 overexpression or knockdown did not significantly alter TRIM33 mRNA levels (Fig 5K), suggesting that SAT1 regulates TRIM33 at the post-translational level. In summary, these results suggest that SAT1 enhances the function of p53 by promoting its acetylation, potentially facilitating the process of ferroptosis.

### SAT1 promotes ferroptosis by enhancing p53 acetylation through TRIM33

**Fig 6:**
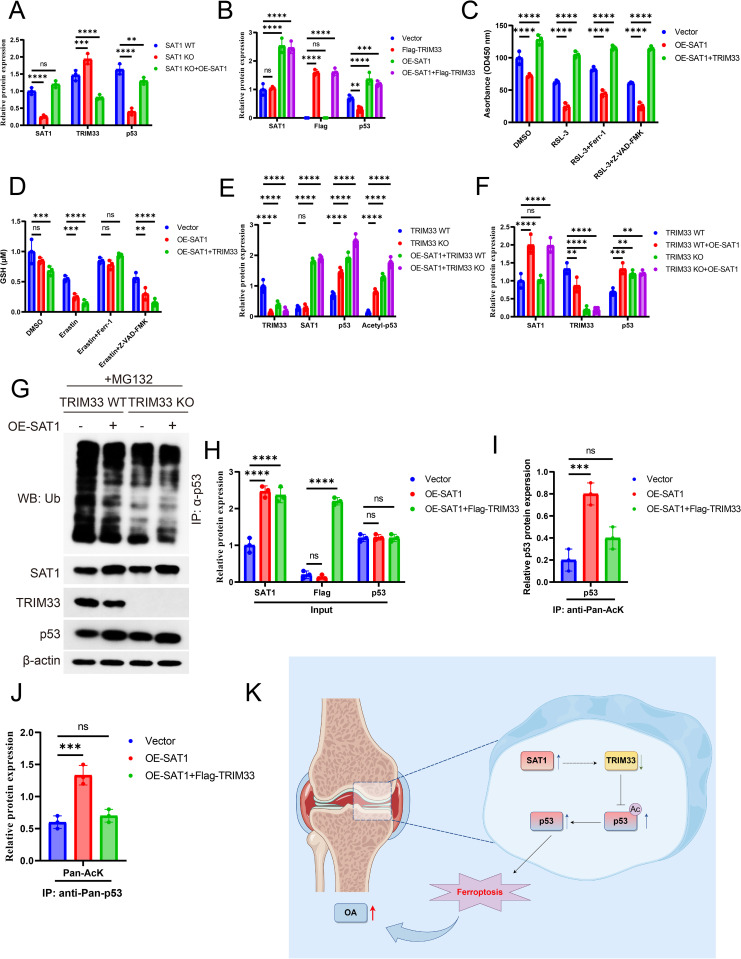
SAT1 promotes ferroptosis by antagonizing the inhibitory effect of TRIM33 on p53 acetylation. (A) Western blot analysis of SAT1, p53 and TRIM33 protein levels in SAT1 WT, SH SAT1 and SH SAT1 with SAT1 transiently expressed ATDC5 cells. (*n* = 3 independent experiments)..(B) Western blot analysis of SAT1, p53 and TRIM33 protein levels in Vector, OE-SAT1, TRIM33 and OE-SAT1 with TRIM33 transiently expressed ATDC5 cells. (*n* = 3 independent experiments). **(C)** CCK8 assay revealed cell viability of Vector, OE-SAT1, and OE-SAT1 with Flag-TRIM33 transiently expressed ATDC5 cells treated with DMSO or RSL3 5 μM or RSL3 5 μM and Ferr-1 5 μM or RSL3 5 μM and Z-VAD-FMK 20 μM for 24 h. (*n* = 3 independent experiments). **(D)** GSH detected assay performed on Vector, OE-SAT1, and OE-SAT1 with Flag-TRIM33 transiently expressed ATDC5 cells treated with DMSO or Erastin 5 μM or Erastin 5 μM and Ferr-1 5 μM or Erastin 5 μM and Z-VAD-FMK 20 μM for 24 h. (*n* = 3 independent experiments).(E) Western blot analysis of SAT1, TRIM33, p53 and acetyl-p53 protein levels in TRIM33 WT and TRIM33 KO ATDC5 cells overexpressed with vector or SAT1. (*n* = 3 independent experiments). **(F)** Western blot analysis of SAT1, TRIM33 and p53 protein levels in TRIM33 WT and TRIM33 KO ATDC5 cells. (*n* = 3 independent experiments).(G) Western blot analysis of SAT1, TRIM33 and p53 protein levels in TRIM33 WT and TRIM33 KO ATDC5 cells with MG132 (20 μM) for 6 h. Lysates from TRIM33 WT and TRIM33 KO ATDC5 cells were subjected to IP assay using anti-ubiquitin antibody. (*n* = 3 independent experiments). **(H-J)** Lysates from Vector, OE-SAT1, and OE-SAT1 with Flag-TRIM33 transiently expressed ATDC5 cells loaded at a ratio of 2:1:2 was subjected to IP assay. (*n* = 3 independent experiments).(K) A model of SAT1-mediated ferroptosis.Error bars represent ± / + SEM unless indicated otherwise. *p < 0.05, **p < 0.01, ***p < 0.001,****p < 0.0001.

Given the interaction between TRIM33 and p53, and the ability of TRIM33 to destabilize p53 by promoting its K48-linked ubiquitination, thereby affecting cellular metabolic processes [31], we speculated that TRIM33 might be involved in SAT1-regulated ferroptosis. Western blot results showed that in SAT1-knockdown cells, the protein expression level of TRIM33 was significantly increased, while the level of p53 protein was significantly decreased; conversely, re-expression of SAT1 reduced the expression level of TRIM33 and restored the level of p53 protein (Fig 6A). Moreover, in cells overexpressing SAT1, exogenous expression of TRIM33 did not affect the level of SAT1 protein but significantly decreased the level of p53 protein (Fig 6B). These results indicate that SAT1 regulates the level of p53 protein through TRIM33. Further experiments revealed that in ATDC5 cells overexpressing SAT1, exogenous expression of TRIM33 significantly enhanced cell viability and intracellular GSH levels (Fig 6C-D). As a positive control, cell death induced by TRIM33 could be antagonized by the apoptosis inhibitor Z-VAD-FMK (Fig 6C-D). By knocking out endogenous TRIM33 in cells using CRISPR-Cas9 and overexpressing SAT1, we found that SAT1 could only increase total p53 protein and acetylated p53 levels in control cells with normal TRIM33 expression, but not in TRIM33-deficient cells (Fig 6E). Subsequently, we overexpressed SAT1 in TRIM33-deficient cells and found that the level of p53 protein no longer increased (Fig 6F). Consistent with previous results in this study, knockdown of TRIM33 inhibited the degradation of p53 protein, while GINS4 inhibited the ubiquitination of p53 in Snail cells (Fig 6G). By overexpressing TRIM33 in SAT1-overexpressing cells and detecting the acetylation level of p53, we found that TRIM33 significantly antagonized the increase in p53 protein and acetylation levels induced by SAT1 overexpression (Fig 6H-J). In summary, our study demonstrates that SAT1 specifically recognizes and promotes the acetylation of p53 through TRIM33, thereby promoting ferroptosis.

## Discussion

In this study, we investigated the role of ferroptosis in the development of OA and its underlying molecular mechanisms. Ferroptosis is a form of iron-dependent programmed cell death that has been closely associated with the occurrence and development of various diseases in recent years [32,33]. Our study identifies a novel regulatory axis involving SAT1, p53, and ferroptosis in the pathogenesis of OA. Rather than simply recounting the experimental outcomes, we propose that the SAT1-p53-ferroptosis pathway represents a critical convergence point for metabolic and stress signaling in chondrocytes. The dysregulation of this axis under inflammatory and mechanical stress conditions may serve as a key driver of cartilage degeneration, positioning it as a potential mechanistic link between upstream triggers and downstream cellular demise in OA. This perspective underscores the pathway’s relevance not only as a contributor to disease progression but also as a promising node for therapeutic intervention.OA is a complex degenerative joint disease, the pathogenesis of which involves a variety of factors, including inflammation, imbalance of extracellular matrix metabolism, and cell death [34,35]. In recent years, ferroptosis, as a novel form of iron-dependent programmed cell death, has been considered to be closely related to the occurrence and development of OA [36]. The main characteristics of ferroptosis are increased levels of intracellular iron ions and intensified lipid peroxidation reactions, leading to cell membrane damage and cell death [36]. In OA, ferroptosis of chondrocytes can lead to the degradation of cartilage matrix and the exacerbation of inflammatory responses, thereby promoting disease progression [37]. SAT1 (spermidine/spermine N1-acetyltransferase 1), a key enzyme in polyamine metabolism, plays an important role in the process of ferroptosis. Studies have shown that the activation of SAT1 is closely related to the upregulation of the expression of ALOX15, a key participant in ferroptosis [38]. In OA models, inhibiting the expression of SAT1 can significantly reduce ferroptosis and inflammatory responses in chondrocytes, indicating that SAT1 may affect the ferroptosis process by regulating the expression of ALOX15, thereby influencing the progression of OA [9]. In this study, we first screened out differentially expressed genes related to ferroptosis, SAT1, through bioinformatics. Then, through cell proliferation experiments, we found that ferroptosis inducers showed a time-dependent effect on ferroptosis. After inducing ferroptosis, SAT1 overexpression promoted ferroptosis characterized by lipid peroxidation and GSH depletion. Next, SAT1 overexpression also induced ferroptosis in the mouse OA model *in vivo*. The observed upregulation of ACSL4 and downregulation of GPX4 upon SAT1 overexpression further support the pro-ferroptotic role of SAT1, as ACSL4 promotes lipid peroxidation while GPX4 acts as a key inhibitor of ferroptosis. Although more experiments are needed to determine the exact role of SAT1 in ferroptosis, our results indicate the role of SAT1 in the development of OA.The stability of the p53 protein is one of the key factors for its normal physiological function. In cells, the stability of p53 is finely regulated by a variety of factors, among which acetylation modification plays a crucial role. Acetylation mainly occurs in the N-terminal transcriptional activation domain of the p53 protein, and this modification can significantly enhance the stability of the p53 protein [39]. When p53 is acetylated, its conformation changes, thereby reducing the degradation of the protein by proteases and extending the half-life of p53 protein in cells [40]. In addition, acetylation may also affect the interaction of p53 with other proteins, further regulating its function in cells [39]. In this study, we observed that SAT1 overexpression increased ferroptosis in a p53-dependent manner both *in vitro* and *in vivo*. We noticed that SAT1 increased the stability of p53 protein because the ubiquitination level of p53 was downregulated by promoting its acetylation level. That is to say, SAT1 can promote the acetylation level of p53. The effect of SAT1 on the stability of p53 protein is related to the acetylation modification sites, which needs further exploration.TRIM33, as an E3 ubiquitin ligase, plays a key role in a variety of biological processes, including DNA repair, cell differentiation, inflammation, and cancer [31]. In esophageal squamous cell carcinoma (ESCC), TRIM33 promotes tumor growth by regulating the K48-linked ubiquitination of p53 [31]. TRIM33 interacts with p53, inhibits its stability, and thereby promotes the expression of its downstream glycolytic target genes, such as GLUT1, HK2, PKM2, and LDHA [31]. Although current research mainly focuses on the effects of TRIM33 on p53 ubiquitination, acetylation modification may also play an important role in the interaction between TRIM33 and p53. Acetylation modification can affect the stability and function of p53, and TRIM33 may regulate its activity by affecting the acetylation state of p53. In this study, we found that in TRIM33 KO cells, SAT1 overexpression did not affect the protein level and acetylation level of p53. Overexpression of TRIM33 in SAT1-overexpressing cells indeed reduced the elevated p53 acetylation level. These results suggest that SAT1 promotes ferroptosis possibly by antagonizing the reduction of p53 acetylation level through TRIM33. Future research can further explore whether TRIM33 regulates its role in apoptosis and tumorigenesis by affecting the acetylation of p53.In summary, this study describes the role of SAT1 in the progression of OA. The upregulation of SAT1 inhibits the expression of TRIM33, which, as an E3 ubiquitin ligase, can affect the stability of the p53 protein through ubiquitination modification, thereby further influencing SAT1’s acetylation of p53. In the context of ferroptosis, the stability of p53 increases, further promoting the occurrence of ferroptosis. This finding suggests that SAT1 may be involved in the regulation of ferroptosis in chondrocytes in OA by modulating the TRIM33-p53 axis. Although we have elucidated the changes in SAT1 in the occurrence of OA and its related pathways from a molecular mechanism perspective, the accuracy and effectiveness of these molecules as diagnostic markers or therapeutic targets have not been fully validated in clinical samples, and there is still a gap before clinical translational application can be achieved.

## Supporting information

S1 File Supplemental table 1. (XLS)

S2 File WB original image. (DOCX)
