# Supplementary figures and images for: SAT1 promotes the progression of OA by regulating TRIM33-mediated p53 acetylation to enhance ferroptosis
Source: PLoS One. 2025 Oct 8;20(10):e0332761. doi: 10.1371/journal.pone.0332761 (PMC12507308; doi:10.1371/journal.pone.0332761)

Figure 1D


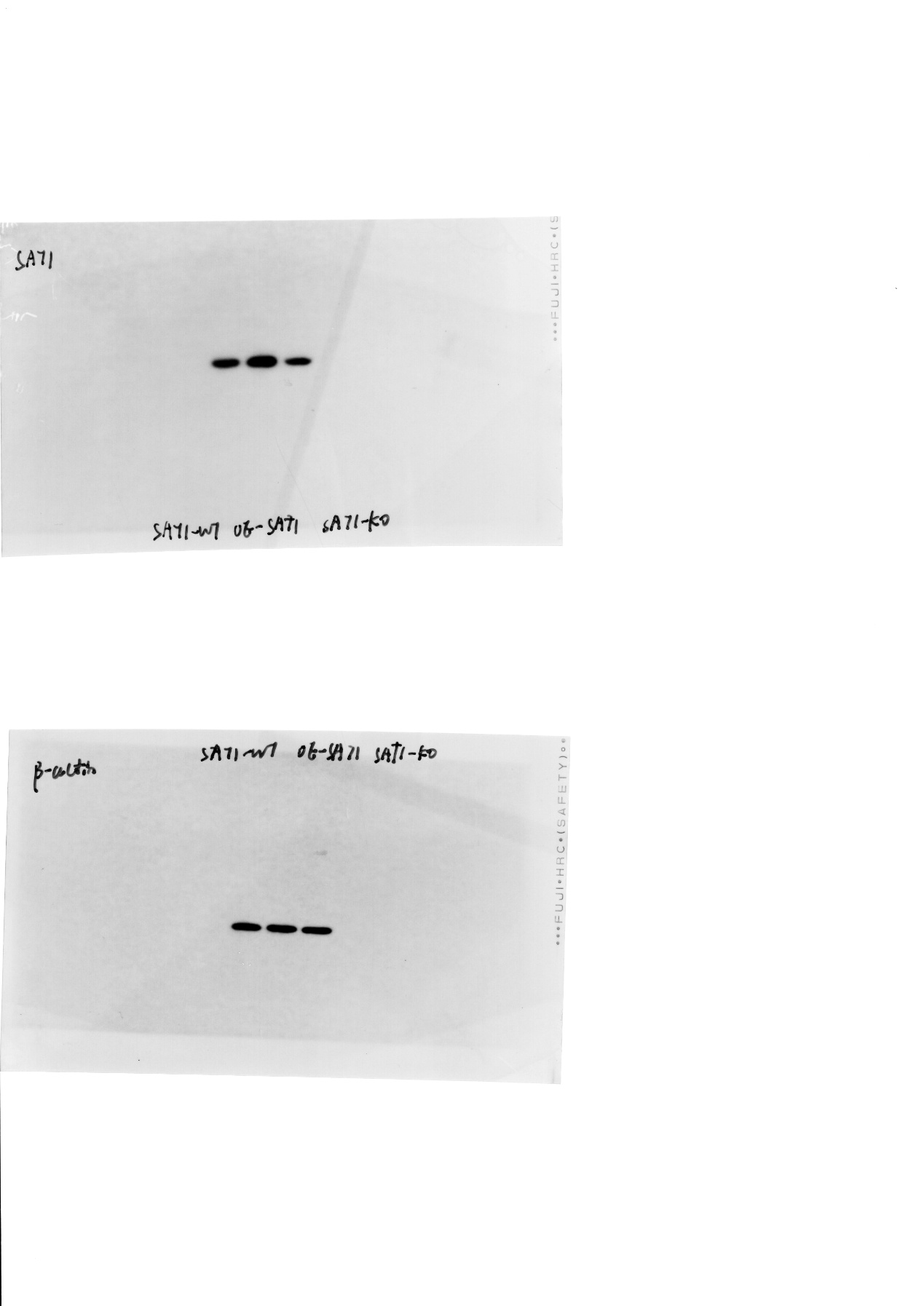


Figure 5E


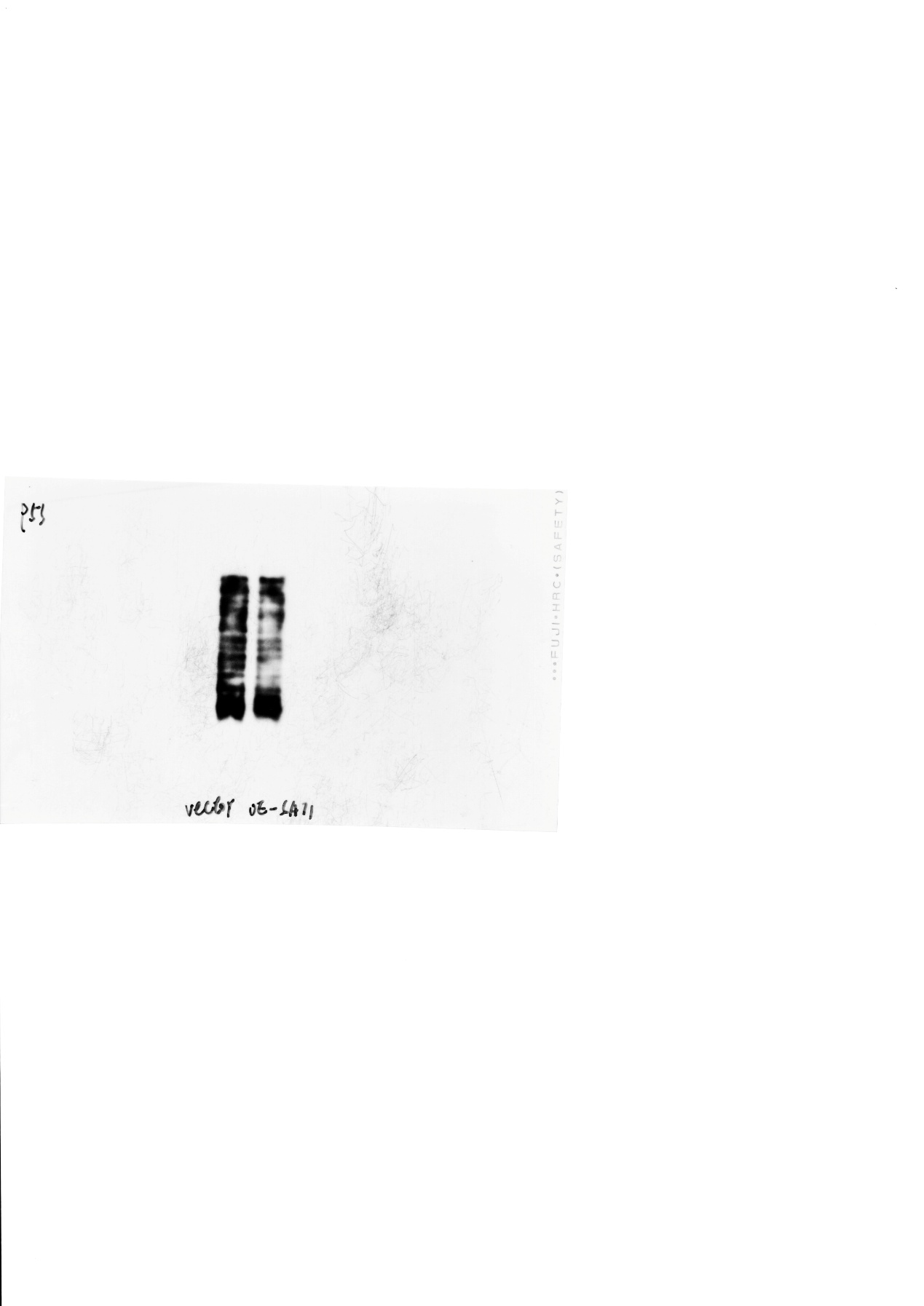


Figure 5F


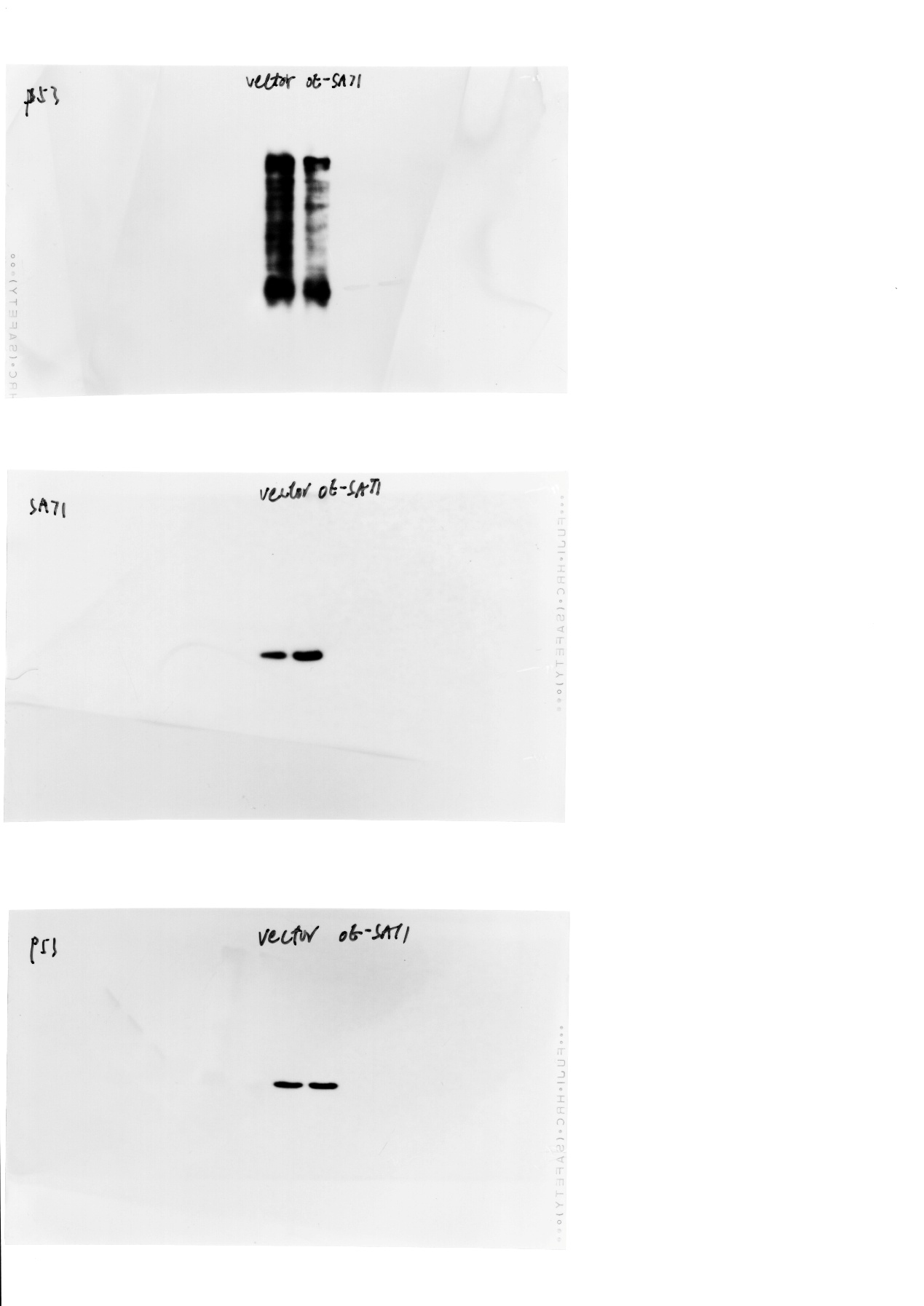


Figure 6G


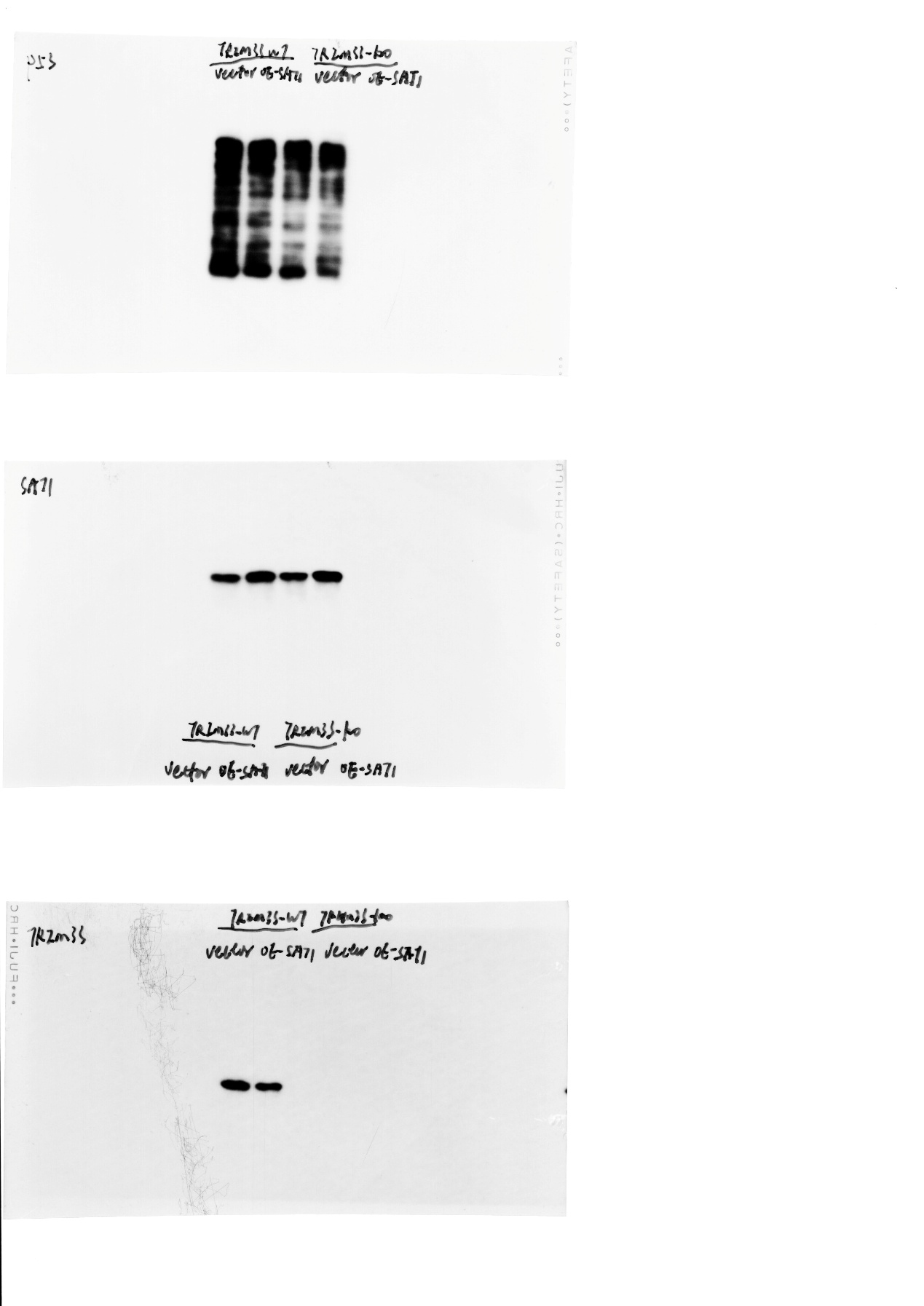


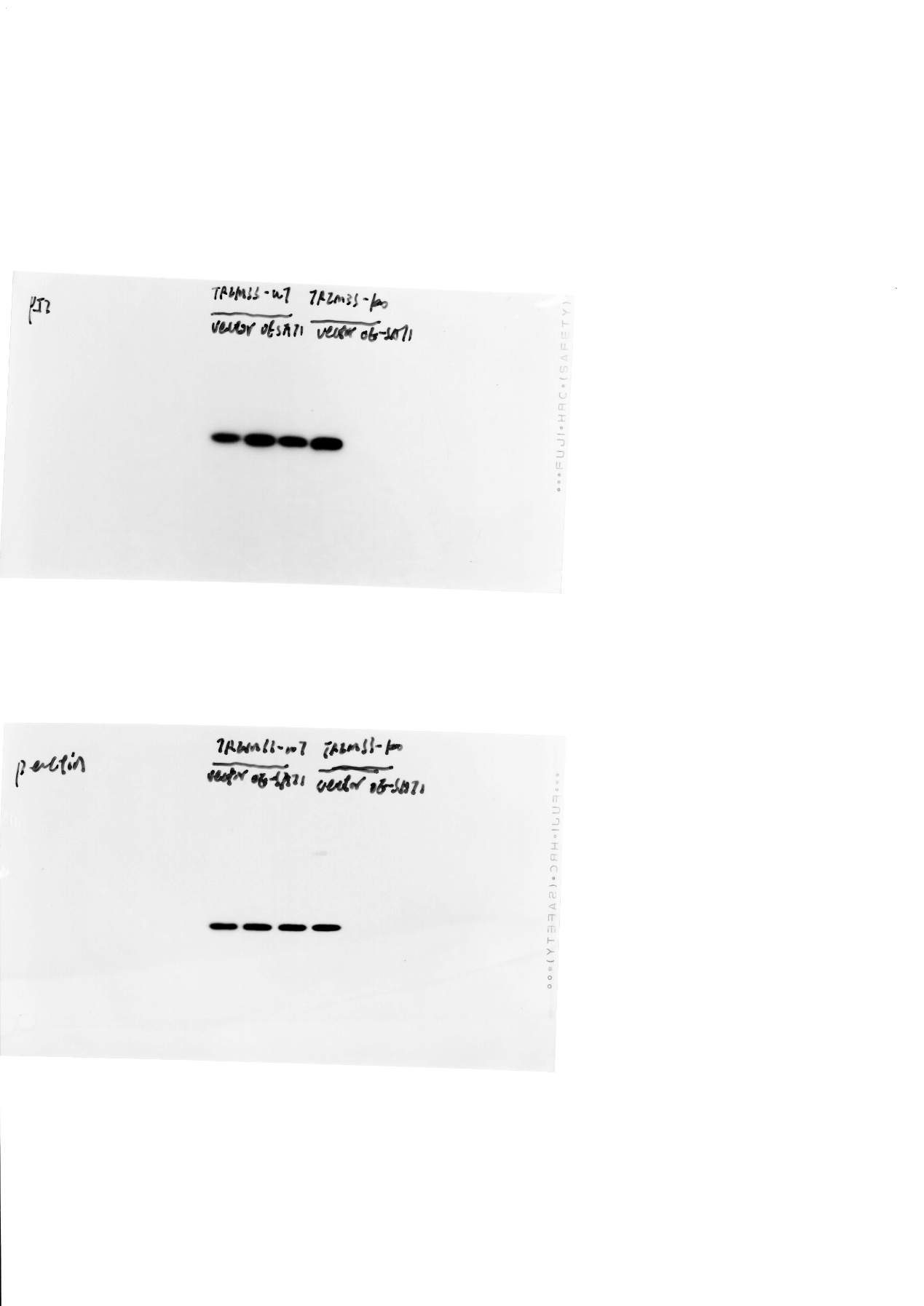

Supplement: S2 File — (DOCX) [file pone.0332761.s002.docx]
